# Bioactive components from *Sinomenium acutum* exhibit anti-inflammatory activity and sensitize cervical cancer cells to paclitaxel

**DOI:** 10.1007/s13659-026-00652-w

**Published:** 2026-10-19

**Authors:** Xilei Wang, Chengjing Zhu, Xinyu Du, Qiang Wang, Jianqing Yu

**Affiliations:** 1 Department of Pharmacy, Zhongnan Hospital of Wuhan University, School of Pharmaceutical Sciences, Wuhan University, Wuhan, 430071 China; 2 Key Laboratory of Combinatorial Biosynthesis and Drug Discovery, Ministry of Education, Wuhan University School of Pharmaceutical Sciences, Wuhan, 430071 China; 3 College of Pharmacy, South-Central Minzu University, 182 Minzu Road, Wuhan, 430074 China

**Keywords:** *Sinomenium acutum* Rehd. et Wils., *Anti-inflammatory*, *Sensitization*, *Bioactive compounds*

## Abstract

Six previously undescribed compounds, together with twelve known compounds, were isolated from *Sinomenium acutum*. The structures of the unreported compounds were determined by comprehensive spectroscopic data analysis, ^13^C NMR calculations followed by DP4 + analysis, ECD calculations, and single-crystal X-ray diffraction analysis. Nine compounds exhibited more potent anti-inflammatory activities than the positive control indomethacin. The structure–activity relationship of the isolates with anti-inflammatory effects was elucidated, providing some useful clues for designing and developing structurally novel anti-inflammatory drugs. Furthermore, five compounds reversed the paclitaxel resistance in paclitaxel-resistant cervical cancer cells HeLa/PTX. The combination of compound **4** and paclitaxel promoted cancer cell apoptosis.

**Graphical Abstract:**

**Figure:**
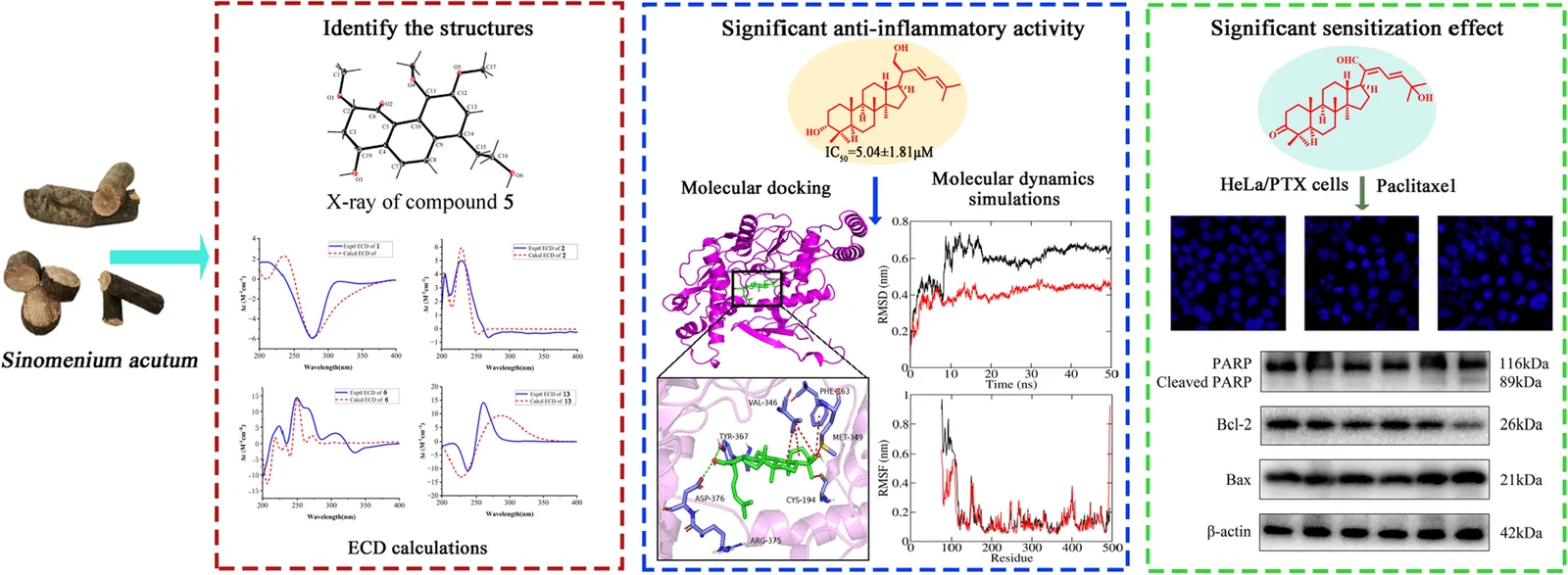

**Supplementary Information:**

The online version contains supplementary material available at https://doi.org/10.1007/s13659-026-00652-w.

## Introduction

*Sinomenium acutum* (Menispermaceae family) is mainly distributed in China [1]. The stems of *S. acutum* are documented in the Chinese Pharmacopoeia for their notable efficacy in treating rheumatic pain, joint swelling, paralysis and cardiovascular disease [2]. Previous research has revealed that *S. acutum* contains different types of compounds, such as alkaloids, triterpenes, and lignans [3]. Sinomenine, the major active alkaloid in *S. acutum*, is the effective principle that has been developed into an important anti-inflammatory drug [4].

Inflammation, a protective mechanism against tissue injury or infection, can contribute to the development of cancer, atherosclerosis, and rheumatoid arthritis when it fails to resolve [5]. Developing safer and more effective drugs remains a challenge due to the complex pathogenesis of inflammation. Natural products, as a critical source of clinical drugs, play a pivotal role in the discovery of anti-inflammatory agents.

Cancer poses a rising threat to global health and has emerged as one of the primary contributors to the global burden of disease [6, 7]. In the past decade, some exceptional breakthroughs have been achieved to combat cancer [8]. However, cancer drug resistance limits therapeutic options and is recognized as a major determinant of treatment failure [9]. Drug resistance arises through diverse mechanisms, involving genetic mutations, tumor microenvironment adaptations, and activation of pro-survival pathways [8, 10]. Cancer drug resistance represents a critical obstacle in clinical therapy. To explore novel strategies to overcome drug resistance, combination chemotherapy has become a promising approach due to multiple drugs with distinct mechanisms of action [9]. Natural products demonstrate significant potential in antitumor therapy [11, 12]. In seeking to identify new drug combinations for targeting drug-resistant cancer cells, we found that several sesquiterpenes synergistically enhance cisplatin efficacy in ovarian cancer cells [13], and some alkaloids isolated from *Piper longum* and *Piper nigrum* sensitize cervical cancer cells to chemotherapeutic agents [14, 15]. Natural medicinal compounds exhibit promising potential for developing innovative strategies to overcome chemotherapy resistance in cancer [16].

**Fig. 1:**
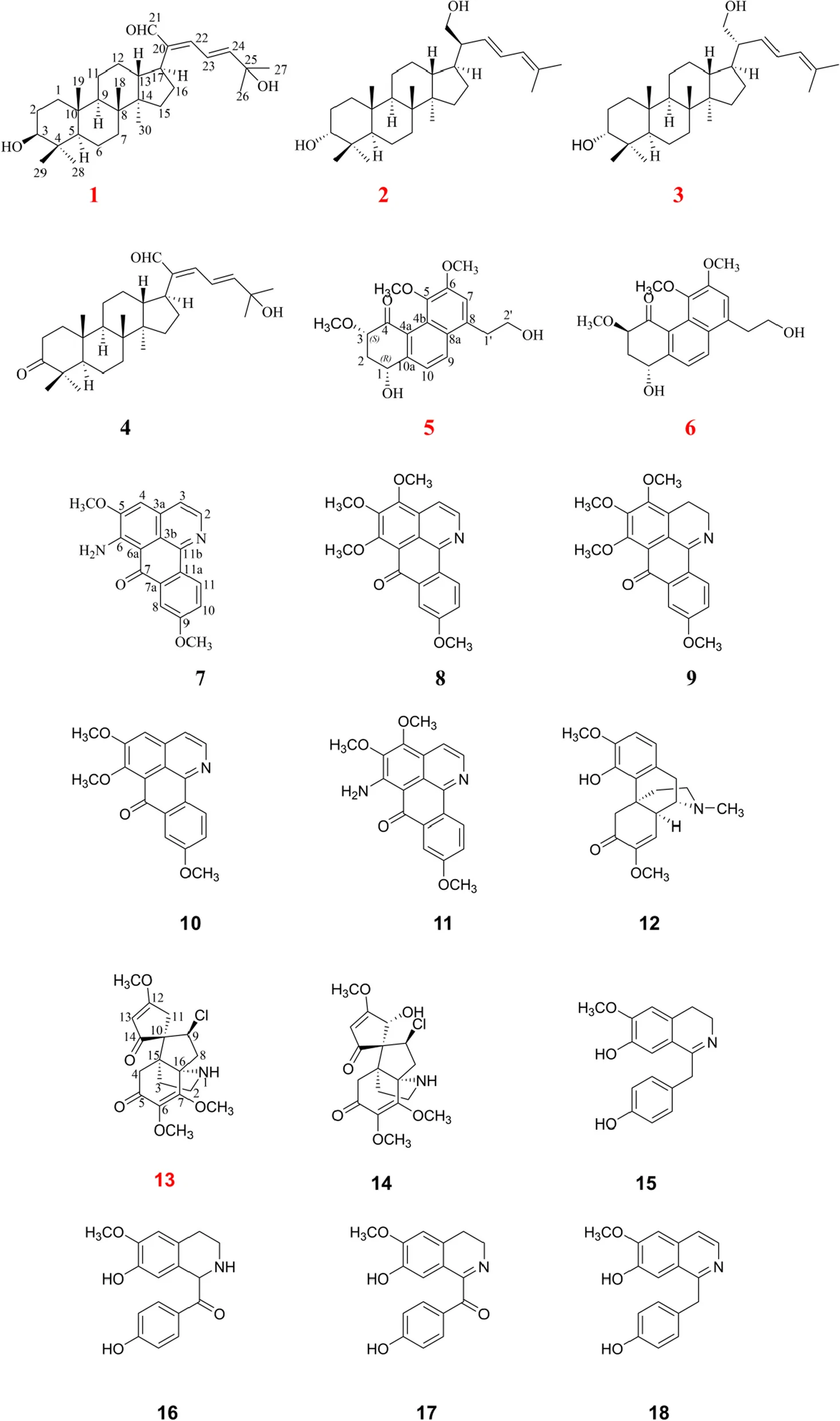
Chemical structures of six unreported compounds (**1**–**3**, **5**, **6**, and **13**) and twelve known ones (**4**, **7**–**12**, **14**–**18**)

Although some studies have shown that *S. acutum* contains a variety of chemical constituents, the bioactive components of this plant have not yet been systematically elucidated. In this work, 18 compounds (Fig. 1), including six new ones, were isolated and characterized, and their anti-inflammatory and chemosensitizing activities to paclitaxel were evaluated. The structure–activity relationship of the isolates was analyzed for their anti-inflammatory effects. This work will promote the further exploration and application of *S. acutum*.

## Results and discussion

### Structural elucidation

**Fig. 2:**
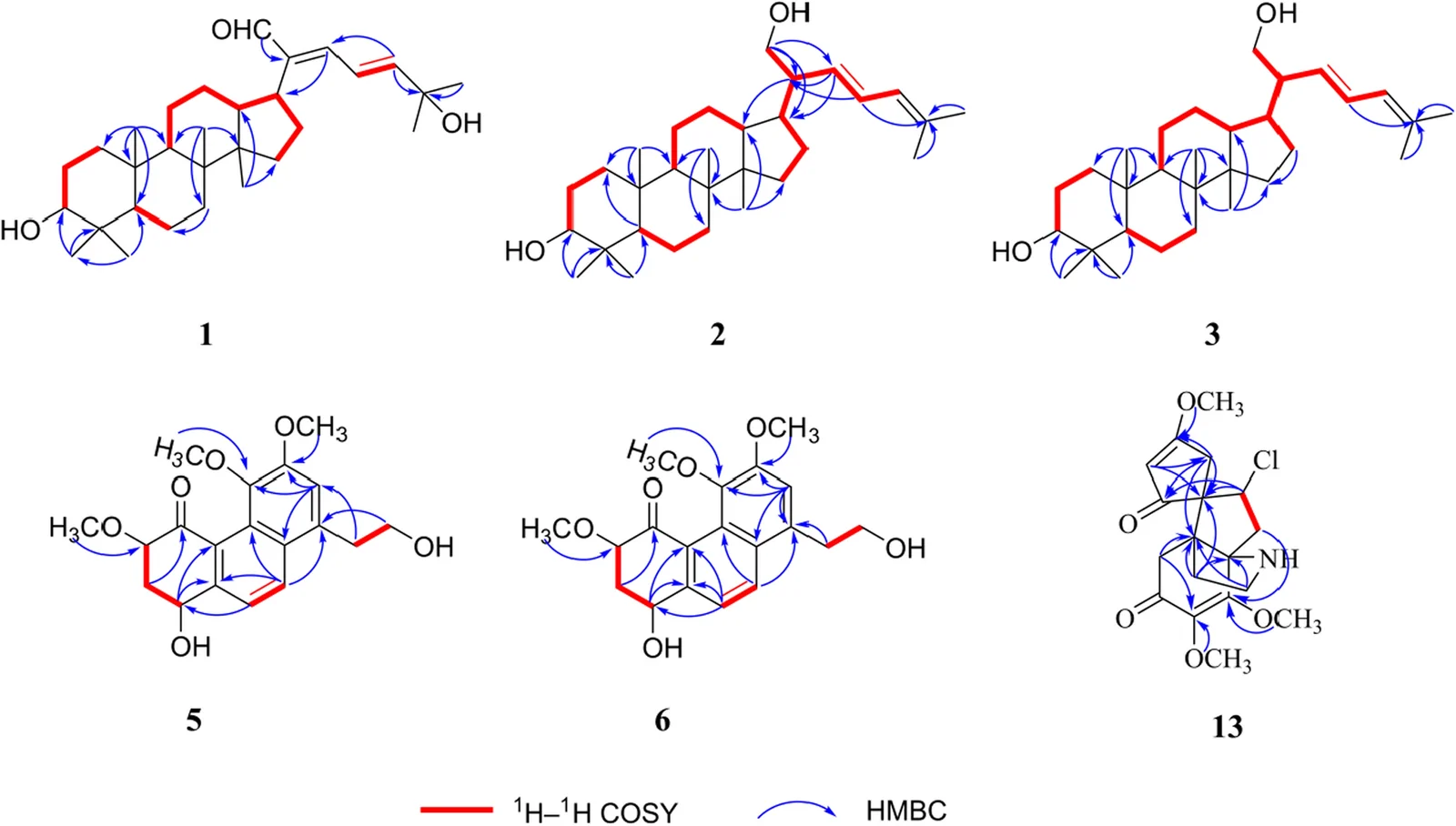
Key ^1^H − ^1^H COSY and HMBC correlations of compounds **1**–**3**, **5**, **6** and **13**

**Fig. 3:**
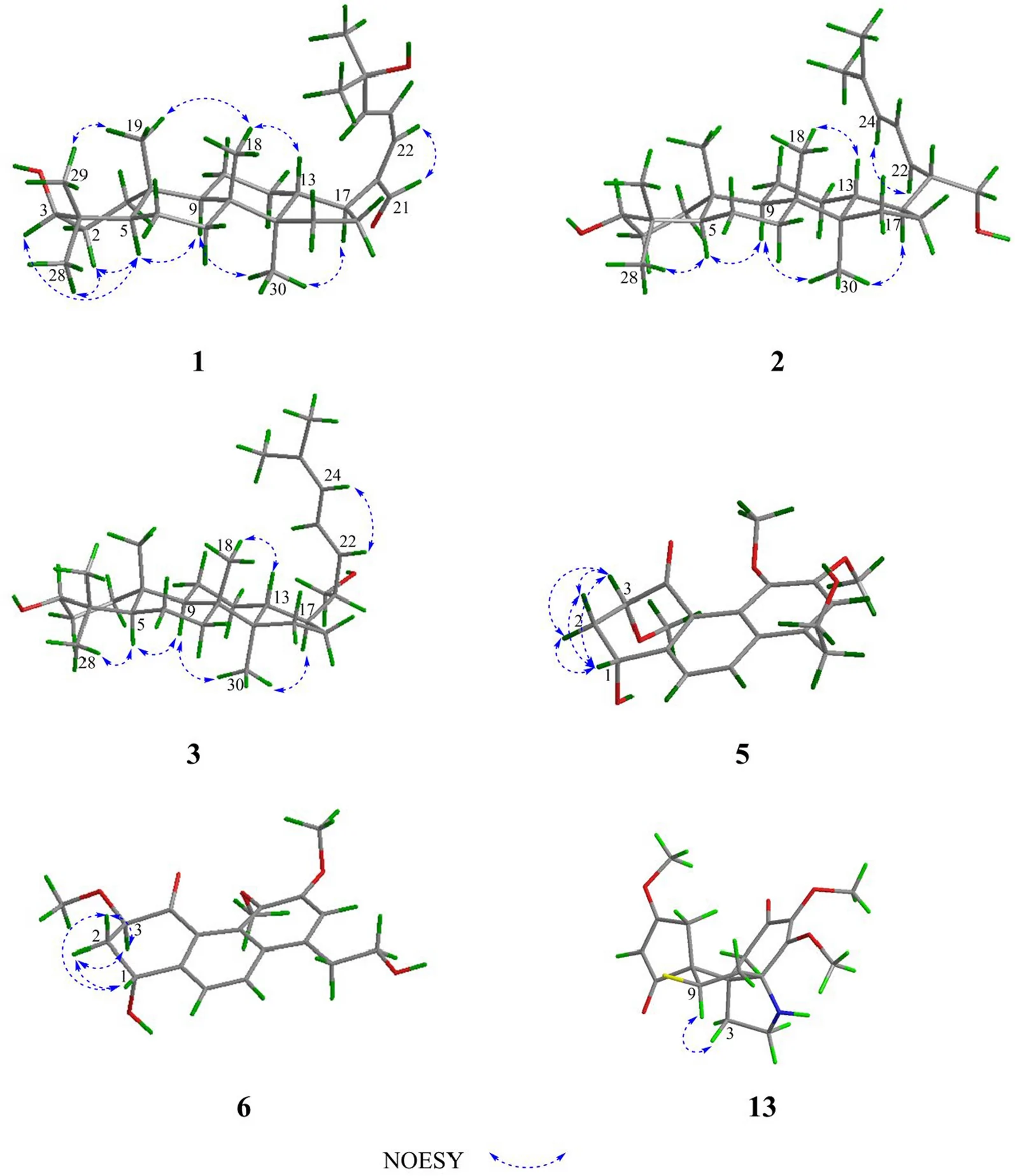
Key NOESY correlations of compounds **1**–**3**, **5**, **6** and **13**

**Fig. 4:**
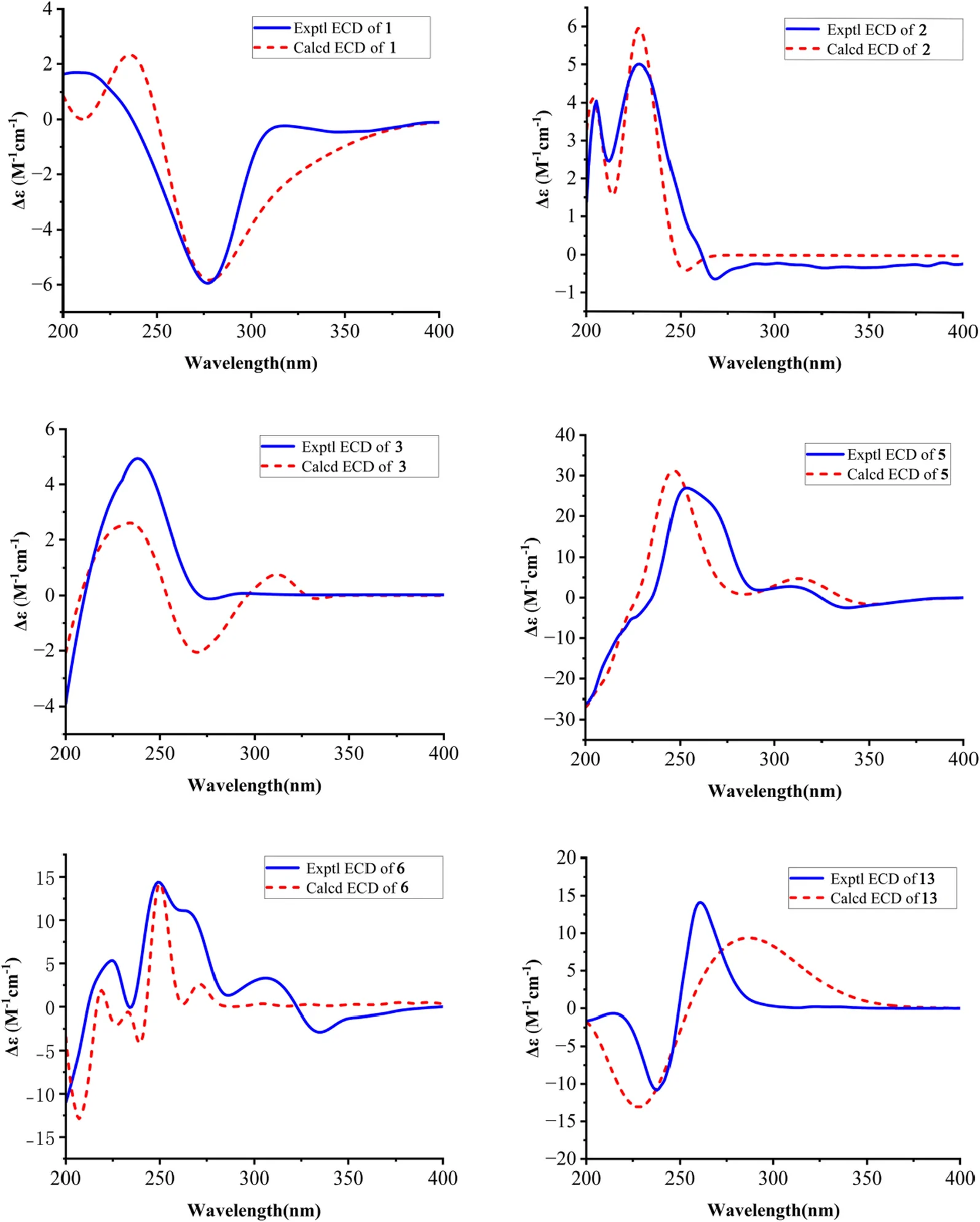
Experimental and the calculated ECD spectra of compounds **1**–**3**, **5**, **6**, and **13**

Compound **1** possessed a molecular formula of C_30_H_48_O_3_, which was supported by the HRESIMS ion peak at *m/z* 479.3488 [M + Na]⁺ (calcd. for C_30_H_48_O_3_Na⁺, 479.3501). The ^1^H NMR data (Table 1) of **1** displayed three olefinic protons [*δ*_H_ 6.77 (1H, H-22), 6.74 (1H, H-23), and 6.28 (1H, d, *J* = 14.3 Hz, H-24)], an aldehyde proton [*δ*_H_ 9.38 (1H, s, H-21)], and seven methyl signals. The ^13^C NMR data (Table 1) and HSQC correlations (Fig. S3) of **1** revealed 30 carbon resonances, including an aldehyde (δC 195.5), four olefinic carbons (*δ*_C_ 144.9, 149.7, 150.8, and 122.0), seven methyls, eight methylenes, five methines (one oxygenated, *δ*_C_ 79.1), and five quaternary carbons (one oxygenated, *δ*_C_ 71.3). The NMR data (Table 1) of **1** were similar to those of coggygrnoid C [17], suggesting they have the same tetracyclic triterpenoid skeleton. The main difference was that the carbonyl at C-3 in coggygrnoid C was substituted by a hydroxyl in **1**, which was also supported by a 2 Da molecular weight difference observed in mass spectrometry. The HMBC correlations (Fig. 2) from Me-28 to C-3 and C-4 corroborated this hypothesis. The NOESY correlations (Fig. 3) of H-3/H-5, H-5/H-9, H-9/Me-30, and Me-30/H-17 indicated that these hydrogens were cofacial and assigned as *α*-oriented, while the correlations of H-13/Me-18, Me-18/Me-19, and Me-19/Me-29 suggested that they were *β*-oriented. Furthermore, both H-5 and Me-28 exhibited NOESY correlations with the same proton (*δ*_H_ 1.57) on C-2 (Fig. 3), thereby supporting the *α*-orientation of Me-28. The NOESY correlation (Fig. 3) of CHO-21/H-22 along with coupling constant (^3^*J*_23,24_ = 14.3 Hz) suggested that the Δ^20(22)^ and Δ^23(24)^ double bonds were both *E* configurations, which confirmed that **1** contained the same side chain as that in coggygrnoid C. Furthermore, the calculated ECD spectrum of (3*S*, 5*R*, 8*R*, 9*R*, 10*R*, 13*R*, 14*R*, 17*S*)−**1** showed a satisfactory match with the experimental ECD spectrum of **1** (Fig. 4), and its absolute configuration was determined. Finally, it was named as sinomenoid A.

**Table 1:** ^1^H (400 MHz) and ^13^C (100 MHz) NMR spectroscopic data of **1**–**3** measured in CDCl_3_

No	1		2		3	
	*δ*_H_ (*J*, Hz)	*δ*_C_	*δ*_H_ (*J*, Hz)	*δ*_C_	*δ*_H_ (*J*, Hz)	*δ*_C_
1	0.94, m, 1H	39.3	1.38, m, 1H	33.7	1.26, m, 1H	33.8
	1.67, m, 1H		1.26, m, 1H		1.41, m, 1H	
2	1.57, m, 1H	27.5	1.92, m, 1H	25.5	1.93, m, 2H	25.5
	1.62, m, 1H		1.54, m, 1H			
3	3.20, dd (11.6, 4.8), 1H	79.1	3.38, m, 1H	76.4	3.38, m, 1H	76.4
4		39.1		37.7		37.7
5	0.73, dd (11.9, 2.3), 1H	56.0	1.23, m, 1H	49.6	1.24, m, 1H	49.7
6	1.44, m, 1H	18.4	1.40, m, 2H	18.3	1.39, m, 2H	18.3
	1.54, m, 1H					
7	1.25, m, 1H	35.7	1.23, m, 1H	35.2	1.22, m, 1H	35.4
	1.59, m, 1H		1.57, m, 1H		1.60, m, 1H	
8		40.7		40.5		40.6
9	1.30, m, 1H	51.0	1.41, m, 1H	50.6	1.41, m, 1H	51.0
10		37.4		37.4		37.4
11	1.10, m, 1H	21.5	1.14, m, 1H	21.3	1.14, m, 1H	21.4
	1.47, m, 1H		1.48, m, 1H		1.53, m, 1H	
12	1.07, m, 1H	25.2	1.09, m, 1H	26.5	1.10, m, 1H	25.4
	1.32, m, 1H		1.73, m, 1H		1.58, m, 1H	
13	2.03, m, 1H	46.5	1.42, m, 1H	45.8	1.45, m, 1H	44.6
14		50.3		50.2		49.5
15	1.20, m, 1H	32.2	1.04, m, 1H	30.9	1.06, m, 1H	31.4
	1.94, m, 1H		1.48, m, 1H		1.43, m, 1H	
16	1.76, m, 1H	27.7	1.30, m, 1H	27.6	1.34, m, 2H	24.2
	1.88, m, 1H		1.81, m, 1H			
17	2.84, m, 1H	37.9	1.51, m, 1H	41.9	1.63, m, 1H	41.2
18	1.01, s, 3H	15.6	0.93, s, 3H	15.7	0.89, s, 3H	15.7
19	0.82, s, 3H	16.4	0.84, s, 3H	16.3	0.84, s, 3H	16.2
20		144.9	2.11, m, 1H	52.7	2.33, m, 1H	48.7
21	9.38, s, 1H	195.5	3.29, m, 1H 3.64, dd, (12.5, 4.1), 1H	64.8	3.43, m, 1H 3.57, dd (12.8, 5.3), 1H	65.6
22	6.77^a^, 1H	149.7	5.30, dd (15.1, 9.6), 1H	132.4	5.35, dd (15.1, 9.3), 1H	129.3
23	6.74^a^, 1H	122.0	6.29, dd (15.1, 10.6), 1H	129.5	6.32, dd (15.1, 10.5), 1H	130.9
24	6.28, d (14.3), 1H	150.8	5.81, d (10.6), 1H	124.8	5.83, d (10.5), 1H	125.0
25		71.3		134.5		134.4
26	1.40, s, 3H	29.8	1.77, s, 3H	26.1	1.77, s, 3H	26.1
27	1.40, s, 3H	29.9	1.75, s, 3H	18.5	1.75, s, 3H	18.5
28	0.77, s, 3H	15.5	0.93, s, 3H	28.5	0.93, s, 3H	28.5
29	0.98, s, 3H	28.2	0.83, s, 3H	22.2	0.83, s, 3H	22.3
30	0.92, s, 3H	15.5	0.84, s, 3H	16.1	0.84, s, 3H	16.3

^a^Signals were overlapped

Compound **2** was determined to possess a molecular formula of C_30_H_50_O_2_, as evidenced by HRESIMS ion at *m/z* 443.3878 [M + H]^+^ (calcd. for C_30_H_51_O_2_^+^, 443.3889). The ^1^H NMR data (Table 1) indicated that there were three olefinic signals [*δ*_H_ 5.30 (1H, dd, *J* = 15.1, 9.6 Hz, H-22), *δ*_H_ 6.29 (1H, dd, *J* = 15.1, 10.6 Hz, H-23), and *δ*_H_ 5.81 (1H, d, *J* = 10.6 Hz, H-24)], seven methyls, one oxygenated methine [*δ*_H_ 3.38 (1H, m, H-3)], and one oxygenated methylene [*δ*_H_ 3.29/3.64 (each 1H, H_2_−21)]. The ^13^C NMR data (Table 1) and HSQC spectra (Fig. S12) of **2** displayed 30 carbon signals, containing four olefinic carbons, seven methyls, nine methylenes (one oxygenated, δC 64.8), six methines (one oxygenated, δC 76.4), and four quaternary carbons. The NMR data of **2** are similar to those of (3*R*, 20*S*)−3,20-dihydroxydammar-24-ene [18], except for the presence of a hydroxymethyl and the absence of a methyl and a hydroxyl in **2**. Additionally, compound **2** generated a Δ^22(23)^ double bond. These changes were also supported by the mass spectrometry-derived molecular weight difference of 2 Da. ^1^H–^1^H COSY correlations (Fig. S14) of H-20/H-22/H-23/H-24 and HMBC correlations from H_2_−21 to C-17, C-20, and C-22, from H-22 to C-17, and C-20, and from H-23 to C-20 support the above conclusions. The ^1^H–^1^H COSY correlation (Fig. S14) of H-20 and H-17, along with the HMBC correlation (Fig. S13) from H-20 to C-13, indicate that the side chain is connected to C-17.

**Fig. 5:**
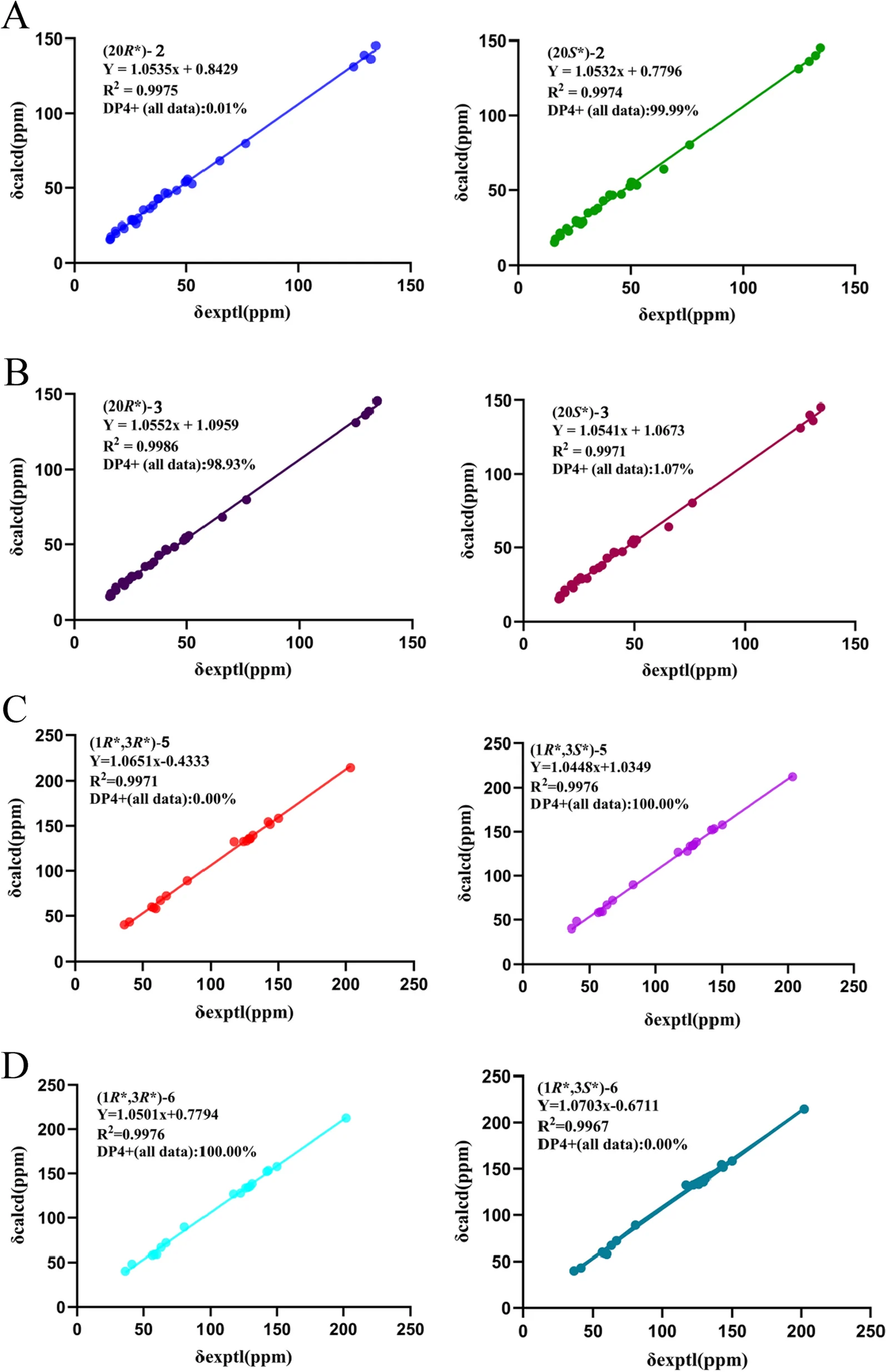
^13^C NMR calculation results of **2**, **3**, **5**, and **6**. **A** The linear correlation curve between the measured and calculated.^13^C NMR chemical shift values of the two potential isomers of compound **2**. **B** The linear correlation curve between the measured and calculated ^13^ C NMR chemical shift values of the two potential isomers of compound **3**. **C** The linear correlation curve between the measured and calculated ^13^ C NMR chemical shift values of the two potential isomers of compound **5**. **D** The linear correlation curve between the measured and calculated ^13^ C NMR chemical shift values of the two potential isomers of compound **6**

The NOESY correlation (Fig. 3) of H-13/Me-18 indicated that these hydrogens have the same orientation and were assigned as *β*-configured. In contrast, the cross-peaks of H-5/H-9, H-5/Me-28, H-9/Me-30, and Me-30/H-17 implied the *α*-orientation of those hydrogens. To define the orientation of 20-H in **2**, NMR calculations with DP4 + analysis were performed. As a result, (20S*)−**2** matched well with the experimental NMR data, with a DP4 + probability of 99.99% (Fig. 5). The coupling constant (^3^*J*_22, 23_ = 15.1 Hz) and NOESY correlation (Fig. 3) of H-22/H-24 revealed the *E* configuration of the Δ^22(23)^ double bond. Furthermore, comparison of the experimental ECD spectrum with the computed ECD curves (Fig. 4) established the absolute configuration of **2** as 3*R, *5*R, *8*R, *9*R, *10*R, *13*R, *14*R, *17*S, *20*S*. Finally, it was named as sinomenoid B.

The molecular formula of compound **3** was identified as C_30_H_50_O_2_ via HRESIMS ion at *m/z* [M + H]^+^ 443.3878 (calcd. for C_30_H_51_O_2_^+^, 443.3889). The ^13^C NMR data of **3** (Table 1) closely resembled those of **2**, except that upfield shifts of 3.4, 4.0, and 3.1 ppm were observed for C-16, C-20, and C-22, respectively, in **3** with respect to **2**.

The presence of the NOESY correlations (Fig. 3) of H-5/H-9, H-5/Me-28, H-9/Me-30, and Me-30/H-17 revealed the *α*-orientation of these hydrogens, while the NOESY correlation of H-13/Me-18 exhibited that they are *β*-configured. NMR calculations combined with DP4 + analysis revealed a 98.93% probability for (20R*)−**3** (Fig. 5), which was used to determine the orientation of 20-H in compound **3**. The NOESY correlation between H-22 and H-24 (Fig. 3), together with the coupling constant (^3^*J*_22, 23_ = 15.1 Hz), revealed the *E* configuration of the Δ^22(23)^ double bond. The experimental ECD spectrum of **3** was consistent with the calculated one (3*R,*5*R,*8*R,*9*R,*10*R,*13*R,*14*R,*17*S,*20*R*)−**3** (Fig. 4)**.** Therefore, the absolute configuration of **3** was defined as 3*R, *5*R, *8*R, *9*R, *10*R, *13*R, *14*R, *17*S, *20*R*. Finally, **3** was named as sinomenoid C.

Compound **5** possessed the molecular formula of C_19_H_22_O_6_ based on its HRESIMS data at *m/z* 347.1485 [M + H]^+^ (calcd. for C_19_H_23_O_6_^+^, 347.1495). The NMR data (Table 2) of **5** were similar to those of the known compound heliophenanthrone [19], except for the appearance of two methoxy groups and a hydroxyethyl group along with the absence of two methyls. The ^1^H–^1^H COSY correlation (Fig. S32) of H_2_−1′/H_2_−2′ and the HMBC correlations (Fig. S31) from OCH_3_−5 to C-5, from OCH_3_−6 to C-6, from H_2_−1′ to C-7, and from H_2_−2′ to C-8 proved this deduction. Furthermore, a methoxy located at C-2 in heliophenanthrone was shifted to C-3 in compound **5**, which was supported by the HMBC correlation (Fig. S31) from OCH_3_−3 to C-3. The NOESY correlation (Fig. 3) of H-1/H-3 indicated these hydrogens were cofacial. To further determine the relative configuration of C-1 and C-3, two possible isomers ((1R*, 3S*)-**5** and (1R*, 3R*)−**5**) were subjected to NMR calculations with DP4 + analysis. The result showed (1R*, 3S*)−**5** had a 100.00% probability (Fig. 5).

**Table 2:** ^1^H (400 MHz) and ^13^C (100 MHz) NMR spectroscopic data of **5** and **6** measured in CDCl_3_

No	5		6	
	*δ*_H_ (*J*, Hz)	*δ*_C_	*δ*_H_ (*J*, Hz)	*δ*_C_
1	4.89, dd (5.4, 2.9), 1H	67.6	5.11, t (5.5), 1H	67.0
2	2.64, ddd (14.4, 5.8, 2.9), 1H	40.2	2.55, dd (6.7, 5.5), 2H	41.3
	2.71, ddd (14.4, 5.4, 3.1), 1H			
3	4.21, dd (5.8, 3.1), 1H	83.1	4.54, t (6.7), 1H	80.7
4		203.5		202.1
4a		129.3		129.8
4b		126.5		126.4
5		142.6		142.4
6		150.2		150.1
7	7.24, s, 1H	117.3	7.20, s, 1H	117.3
8		131.2		131.3
8a		128.3		128.3
9	8.06, d (8.7), 1H	128.5	7.98, d (8.7), 1H	128.3
10	7.40, d (8.7), 1H	124.3	7.34, d (8.7), 1H	122.5
10a		144.0		143.6
1′	3.29, t (6.5), 2H	36.4	3.24, t (6.7), 2H	36.3
2′	3.93, t (6.5), 2H	63.3	3.87, t (6.7), 2H	63.3
3-OCH_3_	3.59, s, 3H	58.3	3.59, s, 3H	58.3
5-OCH_3_	3.98, s, 3H	59.8	3.96, s, 3H	60.0
6-OCH_3_	3.98, s, 3H	56.7	3.95, s, 3H	56.7

**Fig. 6:**
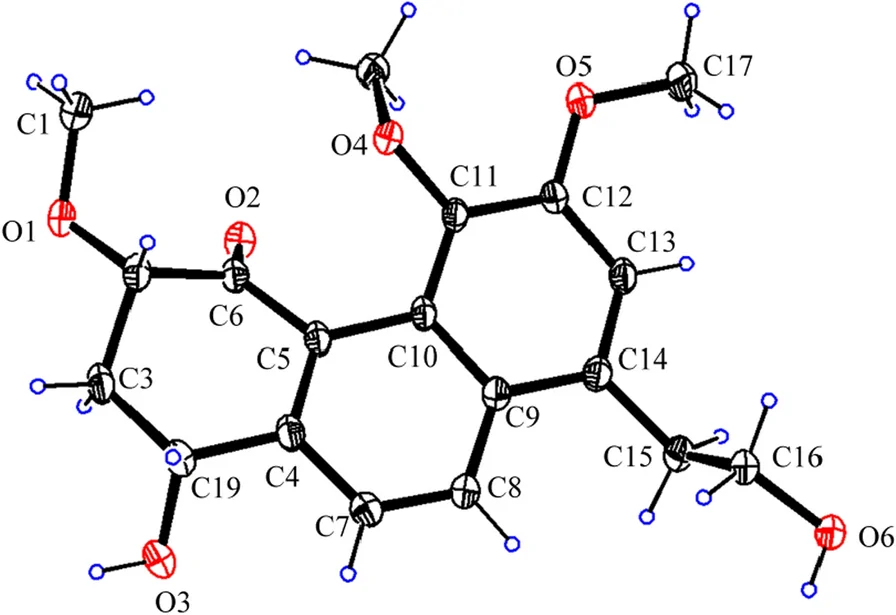
X-ray ORTEP drawing of compound **5**

Furthermore, the absolute configuration of **5** was assigned as 1*R*, 3*S* by comparison of its calculated and experimental ECD spectra (Fig. 4). Subsequently, this assignment was unambiguously confirmed by single-crystal X-ray diffraction analysis (Fig. 6). Accordingly, it was identified as (1*R*, 3*S*)−2, 3-dihydro-1-hydroxy-3, 5, 6-trimethoxy-8-(2′-hydroxyethyl)−4(1*H*)-phenanthrenone*.*

Compound **6** had a molecular formula of C_19_H_22_O_6_, as supported by HRESIMS at *m/z* 369.1304 [M + Na]^+^ (calcd. for C_19_H_22_O_6_Na^+^, 369.1314). The ^1^H and ^13^C NMR data (Table 2) of **6** exhibited high similarity to those of **5**, except that C-3 (*δ*_*C*_ 80.7) was shifted upfield compared to that in **5** (*δ*_*C*_ 83.1, C-3), implying that compounds **5** and **6** could be stereoisomers. Furthermore, NMR calculations combined with DP4 + analysis revealed a 100.00% probability for (1R*, 3R*)−**6** (Fig. 5). The absolute configuration of **6** was identified as 1*R*, 3*R* by comparing the calculated and experimental ECD spectra (Fig. 4). Finally, it was identified as (1*R*, 3*R*)−2, 3-dihydro-1-hydroxy-3, 5, 6-trimethoxy-8- (2′-hydroxyethyl)−4 (1*H*)-phenanthrenone*.*

Compound **13** possessed a molecular formula of C_18_H_22_ClNO_5_, based on its HRESIMS peaks at *m/z* 368.1254 [M + H]^+^ (calcd. for C_18_H_23_ClNO_5_^+^, 368.1265). The presence of a chlorine atom in **13** was inferred from the 3:1 ratio of [M + H]^+^ to [M + H + 2]^+^ in the mass spectrum [20]. The ^1^H NMR data (Table 3) of **13** displayed three methoxy groups [*δ*_H_ 3.62 (3H, s, 6-OCH_3_), 4.09 (3H, s, 7-OCH_3_), and 3.88 (3H, s, 12-OCH_3_)], and one olefinic proton [*δ*_H_ 5.30 (1H, s, H-13)]. The ^13^C NMR data (Table 3) and HSQC (Fig. S48) indicated 18 carbon resonances categorized to four olefinic carbons (*δ*_C_ 136.8, 163.9, 192.6, and 106.3), two carbonyl groups (*δ*_C_ 194.3 and 205.9), three methoxy groups (*δ*_C_ 60.9, 61.7, and 59.9), five methylenes, one methine, and three quaternary carbons. The NMR data (Table 3) of **13** closely resembled those of dauricumidine (**14**) [21], except for the absence of a hydroxyl group and the upfield shift of C-11 (*δ*_C_ 37.3) in **13**. The HMBC correlations (Fig. S49) from H_2_−11 to C-10, C-12, C-14, and C-15 further proved the above deduction. In the NOESY spectrum (Fig. S51), the correlation of H-9 with H-3*β* suggested an *α*-configuration for H-9. The calculated ECD spectrum of (9*S*, 10*S*, 15*R*, 16*R*)−**13** was consistent with the experimental one (Fig. 4). Therefore, the absolute configuration of **13** was assigned as 9*S*, 10*S*, 15*R*, 16*R.* Finally, **13** was named as 11-dehydroxy-dauricumidine.

**Table 3:** ^1^H (400 MHz) and ^13^C (100 MHz) NMR spectroscopic data of **13** measured in CD_3_OD

No	*δ*_H_ (*J*, Hz)	*δ*_C_
2	2.89, m, 1H	44.6
	2.77, m, 1H	
3	1.81, m, 1H	43.1
	1.68, m, 1H	
4	2.37, d (17.8), 1H	44.8
	2.27, d (17.8), 1H	
5		194.3
6		136.8
7		163.9
8	2.73, m, 1H	47.4
	2.31, m, 1H	
9	4.40, dd, (12.0, 7.1), 1H	60.8
10		66.0
11	2.82, d (18.2), 1H	37.3
	2.68, d (18.2), 1H	
12		192.6
13	5.30, s, 1H	106.3
14		205.9
15		53.6
16		71.7
6-OCH_3_	3.62, s, 3H	60.9
7-OCH_3_	4.09, s, 3H	61.7
12-OCH_3_	3.88, s, 3H	59.9

The known compounds (**4**, **7**–**12**, **14**, and **15**–**18**) were identified as coggygrnoid C (**4**) [17], sinocutumine J (**7**) [2], bianfugenine (**8**) [22], 2, 3-dihydrodauriporphine (**9**) [23], menisporphine (**10**) [24], daurioxoisoporphine B (**11**) [25], sinomenine (**12**) [25], dauricumidine (**14**) [21], 7-hydroxy-6-methoxy-1-(4-hydroxybenzyl)−3,4-dihydroisoquinoline (**15**) [26], 1-(4-hydroxybenzoyl)−6-methoxy-1,2,3,4-tetrahydroisoquinolin-7-ol (**16**) [27], longifolonine (**17**) [28], and juzirine (**18**) [28] by comparing of their NMR data with those reported in the literature.

### Compounds exhibited anti-inflammatory effects

NO is an important chemical transmitter involved in the regulation of numerous key physiological processes [29]. The anti-inflammatory activities of the isolates were assessed by measuring NO production inhibition via the Griess assay, using indomethacin as the positive agent. Compounds **1** and **2** demonstrated excellent anti-inflammatory effects. Nine compounds (**1**, **2**, **4**, **5**, **8**, **9**, **11**, **13**, and **17**) showed anti-inflammatory activities superior to that of indomethacin (Table 4).

**Table 4:** IC_50_ values of NO inhibition of compounds **1**–**18**

Compounds	IC_50_ (μM)	Compounds	IC_50_ (μM)
1	7.18 ± 0.80	10	46.42 ± 3.97
2	5.04 ± 1.81	11	17.88 ± 1.26
3	75.58 ± 3.20	12	41.99 ± 2.88
4	14.14 ± 0.79	13	32.79 ± 4.59
5	15.38 ± 0.70	14	44.89 ± 3.04
6	> 80	15	51.04 ± 1.09
7	51.65 ± 2.81	16	> 80
8	32.88 ± 1.04	17	32.78 ± 1.72
9	25.66 ± 1.96	18	62.32 ± 2.95
Indomethacin	33.01 ± 2.46		

### Molecular docking and MD simulations

**Fig. 7:**
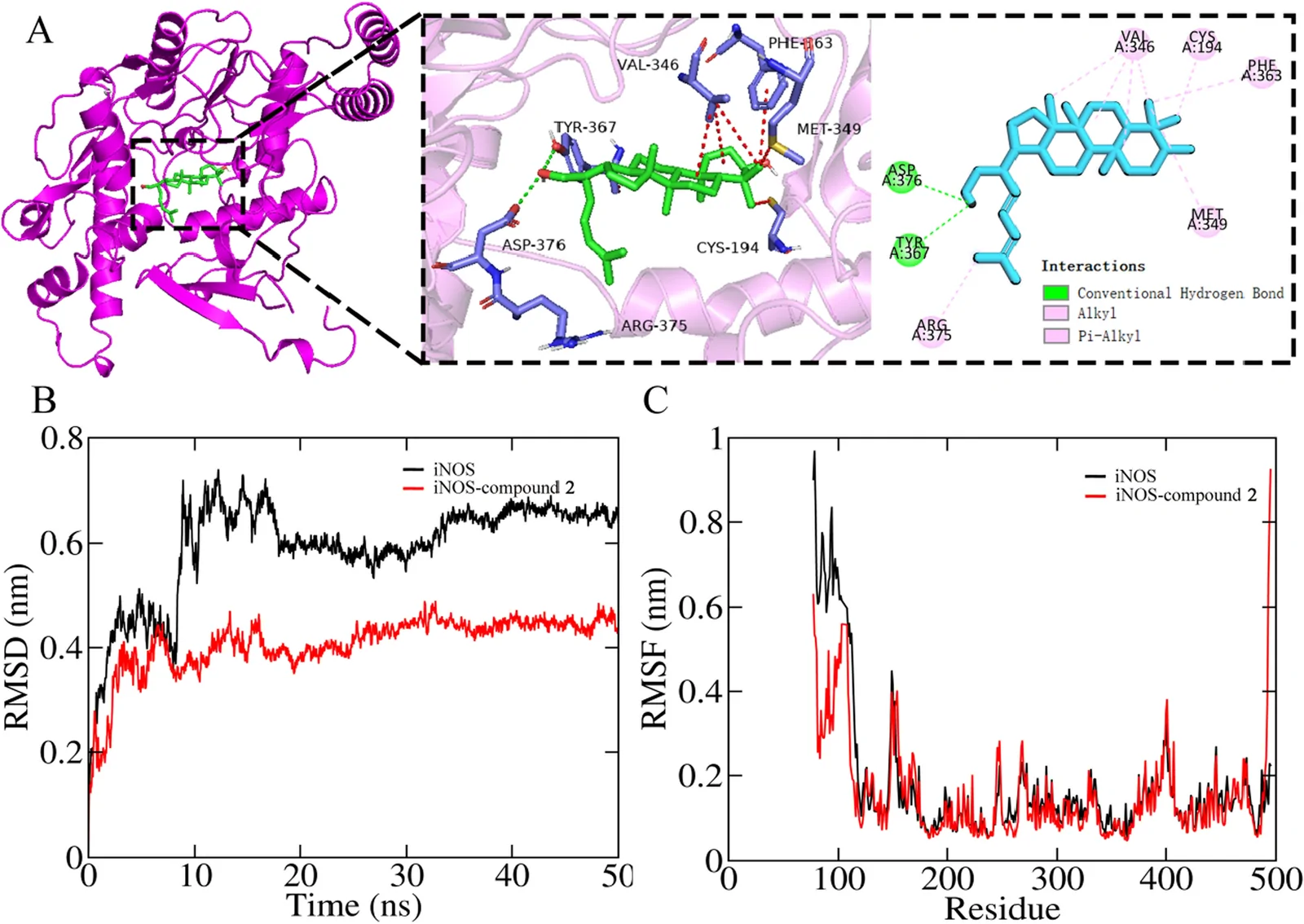
Molecular docking and MD simulation of iNOS with **2**. **A** 2D and 3D binding patterns of compound **2** and iNOS. **B** RMSD values of iNOS with and without compound **2**. **C** RMSF values of iNOS with and without compound **2**

Compound **2** was found to be the most potential anti-inflammatory agent among the tested compounds. The binding energy of iNOS to compound **2** was −9.0 kcal/mol. As shown in Fig. 7A, within the iNOS binding pocket: (i) the C-21 hydroxyl group formed hydrogen bonds with the ASP376 and TYR367 residues; (ii) the two six-membered rings showed hydrophobic interactions with the VAL346 residue; (iii) the C-26 formed an alkyl interaction with the ARG375 residue, C-28 with the CYS194 residue, C-29 with the VAL346, MET349, and PHE363 residues, and C-30 with the VAL346 residue.

The stability of the iNOS–compound **2** complex was evaluated using molecular dynamics simulations and by calculating the RMSD and RMSF parameters. As shown in Fig. 7B, approximately 18 ns after the start of the simulation, the RMSD value changed very little, indicating that the system had reached an equilibrium state. The average RMSD value of the iNOS-compound **2** complex was lower than that of free iNOS, suggesting that iNOS became more stable after binding to **2**. As shown in Fig. 7C, the flexibility of residues in several regions, including residues 180–200, 340–350, and 360–380, was noticeably reduced in the presence of compound **2**.

### Structure–activity relationship analysis of anti-inflammatory compounds

Analysis of the relationship between the anti-inflammatory activity and structures of **1**–**4**, **7**–**11**, and **15**–**18** revealed the following structure–activity relationships: (i) the 3-OH and 20*α*-H groups act as critical structural elements in triterpenoids; (ii) the 4-OCH_3_ and 6-NH_2_ groups in aporphine alkaloids may serve as essential functional groups; (iii) the C = N and C = O double bonds are recognized as key bioactive groups in benzyl tetrahydroisoquinoline alkaloids.

Among triterpenoids, compound **4** with a 3-C = O group showed weaker anti-inflammatory effect than compound **1** with 3-OH. Meanwhile, compound **2**, possessing a 20*α*-H, exhibited stronger anti-inflammatory effect than **3** with a 20*β*-H. Among aporphine alkaloids, compound **7** without a 4-OCH_3_ group showed weaker anti-inflammatory activity than **11**, indicating that the 4-OCH_3_ group contributes more to the anti-inflammatory effects. Compound **8** with a 4-OCH_3_ group showed more potent anti-inflammatory activity than **10**, further supporting the essential role of 4-OCH_3_ in the anti-inflammatory activity. Compound **11** with a 6-NH_2_ exhibited stronger anti-inflammatory activity than **8** with a 6-OCH_3_, suggesting that 6-NH_2_ may be more effective in enhancing anti-inflammatory activity than 6-OCH_3_. Compound **8** with a Δ^2(3)^ double bond exhibited weaker anti-inflammatory activity than **9**. Among benzyl tetrahydroisoquinoline alkaloids, compound **15** without Δ^3(4)^ double bond possessed more potent anti-inflammatory activity than **18** with a Δ^3(4)^ double bond, which further supported the Δ^3(4)^ double bond was a deactivating group to the anti-inflammatory activity. Compound **17** with a 11-C = O group exhibited more significant anti-inflammatory activity than **15**, which indicated that 11- C = O group may be an activating group. Compound **16**, with a C-N single bond between positions 1 and 2, exhibited weaker anti-inflammatory activity than **17** containing a C = N double bond at the same positions, demonstrating that the C = N double bond contributed more to the anti-inflammatory activity.

### Compounds sensitized HeLa/PTX cells to paclitaxel

**Fig. 8:**
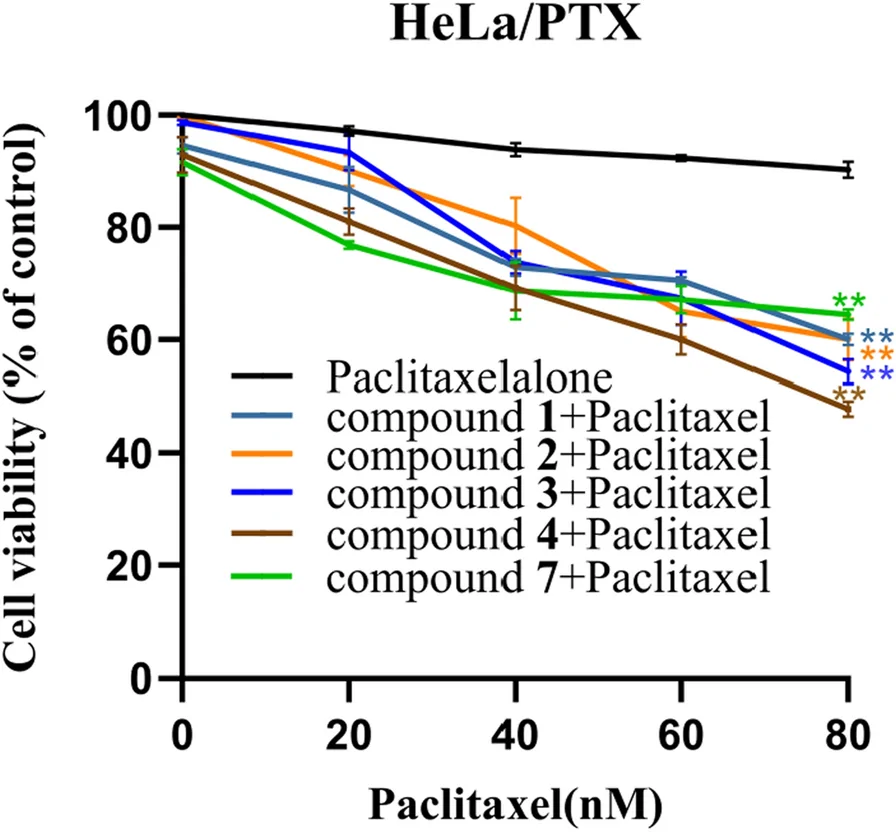
Effect of combination treatment of compounds (**1**–**4**, **7**) and paclitaxel on cell viability. HeLa/PTX cells were treated with paclitaxel alone or in combination with the compounds (50 μM) for 72 h. The Tukey test was used to compare the cell viability in the combination treatment with that of paclitaxel treatment alone. The asterisks indicate a significant difference (** *p* < 0.01)

No significant reduction in cell viability was observed with increasing concentrations of paclitaxel alone, demonstrating resistance of HeLa/PTX cells to paclitaxel, as presented in Fig. 8. Notably, co-treatment with paclitaxel and compounds (**1**–**4**, **7**) resulted in a significant reduction in cell viability.

### Combination treatment inhibited cell proliferation

**Fig. 9:**
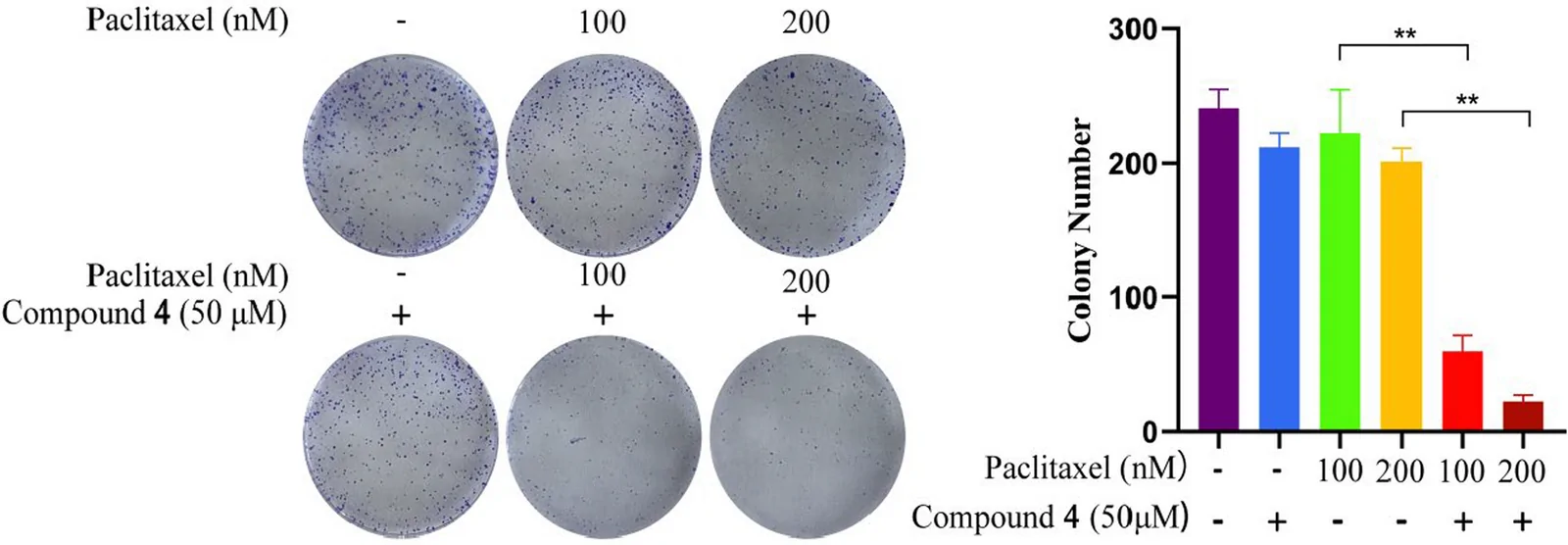
Cell colony formation in HeLa/PTX cells treated with paclitaxel alone or in combination with 4 for 12 h. Data are shown as the mean ± SD (n = 3). The asterisks indicate a significant difference (** *p* < 0.01)

Compound **4** was selected for further analysis. As illustrated in Fig. 9, co-treatment with compound **4** and paclitaxel markedly inhibited colony formation.

### Combination treatment induced apoptosis in HeLa/PTX cells

**Fig. 10:**
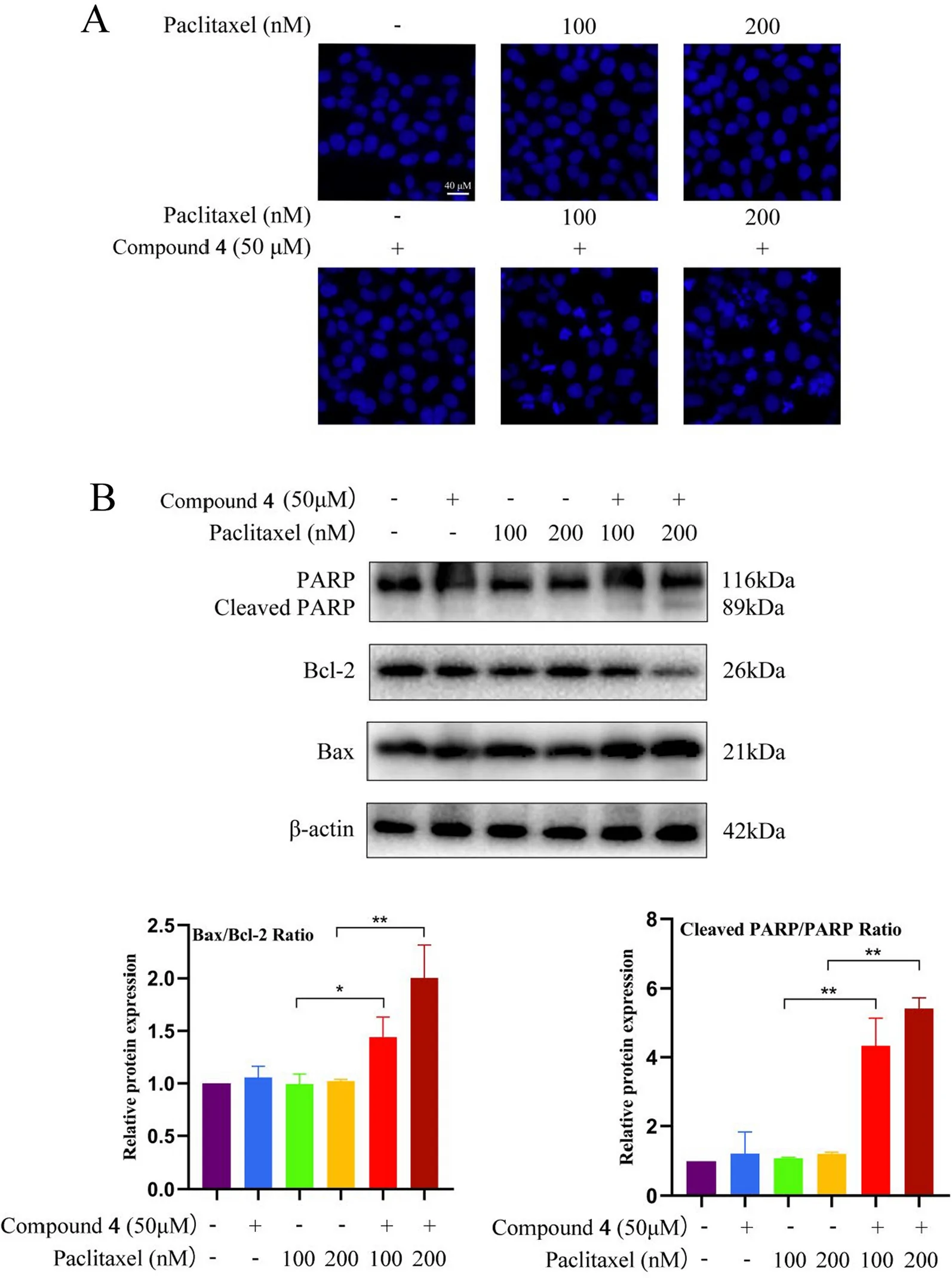
Apoptotic effect of the combination of **4** and paclitaxel on HeLa/PTX cells. The cells were treated with paclitaxel alone or in combination with **4** for 24 h. **A** Nuclear morphology analysis by Hoechst 33,258 staining (scale bar = 40 μm). **B** The expression levels of apoptosis-related genes were analyzed by the western blot assay. The asterisks indicate a significant difference (* *p* < 0.05 and ** *p* < 0.01)

We analyzed nuclear morphology to investigate whether compound **4** enhanced paclitaxel-induced apoptosis in HeLa/PTX. No significant effects were observed with treatment using compound **4** alone or paclitaxel alone in HeLa/PTX cells, but their combination clearly induced apoptotic bodies (Fig. 10A). These findings suggest that the combined treatment exerts anti-tumor effects by inducing apoptosis.

### Combination treatment changed the expression of apoptosis-associated proteins

PARP, a key biomarker of apoptosis, exhibited enhanced cleavage upon combined treatment with **4** and paclitaxel. A significant increase in the Bax/Bcl-2 ratio was induced by the combination of compound **4** and paclitaxel, serving as an important factor for apoptosis in HeLa/PTX cells (Fig. 10B). These findings further demonstrate that the combination treatment promoted apoptosis in HeLa/PTX cells.

## Conclusion

In summary, six new compounds, including three triterpenoids (**1**–**3**), two phenanthrenes (**5** and **6**), and one alkaloid (**13**), were obtained from the stems of *S. acutum*. Five compounds (**11**, **15**–**18**) were described from this plant for the first time. Bioactivities of all compounds (**1**–**18**) obtained were assessed. Nine compounds showed significantly stronger anti-inflammatory activities than the positive control indomethacin. Four triterpenoids and one alkaloid evidently enhance paclitaxel effect against cancer cells. Furthermore, the combination of compound **4** and paclitaxel significantly facilitated apoptosis in cervical cancer cells. These results broaden the structural diversity of *S. acutum* and provide critical insights for developing the novel anti-inflammatory drugs and sensitizing agents against cancer.

## Materials and methods

### General methods

A Nexus 470 FT-IR spectrometer was utilized to acquire the IR spectra. UV spectra were obtained on a Shimadzu UV-1700. Bruker BIOSPIN AVANCE 400 MHz and 600 MHz spectrometers were employed to record the NMR spectra. ECD data were recorded on a JASCO J-810 spectrometer. HRESIMS spectra were acquired using a Thermo Fisher LTQ Orbitrap XL spectrometer.

### Plant material

The stems of *S. acutum* were collected in Hubei Province, China, in 2021, and authenticated by Prof. Jianqing Yu of the School of Pharmaceutical Sciences at Wuhan University.

### Extraction and isolation

The stems of *S. acutum* (8 kg) were immersed in 25 L of 90% ethanol at room temperature to yield 700 g of crude extract, which was subjected to a DA-201 macroporous resin column using a 40–100% methanol (MeOH) in H_2_O gradient, affording four fractions (Fr.1–Fr.4). Column chromatography (CC) was performed on Fr. 2 (8 g) using a gradient system of PE, DCM, and EA (PE: DCM, 1:6; DCM: EA, 50:1–0:1), yielding Fr.2.1–Fr.2.9. Fr.2.4.1 to Fr.2.4.7 were obtained by subjecting Fr.2.4 to an ODS column (MeOH–H_2_O, 30–100%). Purifying Fr.2.4.1 repeatedly by CC over silica gel (DCM:MeOH, 22:1) yielded **12** (20 mg), **14** (8 mg), and **17** (14 mg). Purification of Fr.2.4.2 by CC over silica gel, eluting with DCM–MeOH (20:1), yielded **13** (60 mg). **5** (7 mg, t_R_ = 15 min) and **6** (8 mg, t_R_ = 10.5 min) were obtained by purifying Fr.2.4.3 via semipreparative HPLC. Fr.2.5 was subjected to an ODS column (MeOH–H_2_O, 30–100%) and then further isolated by silica gel CC (DCM: MeOH, 13:1) to yield **15** (37 mg) and **16** (13 mg). Silica gel CC (DCM: MeOH, 12:1) was used to isolate compound **18** (14 mg) from Fr. 2.6.7. Silica gel column chromatography was applied to purify Fr.3 (14 g) using eluents of PE: DCM (1:4) and DCM:MeOH (30:1–0:1), yielding Fr.3.1–Fr.3.9. Compound **10** (4 mg) was isolated by recrystallization from Fr.3.2.6 in a methanol. Purification of Fr.3.2.8 via silica gel CC with PE: DCM (4:1) as the eluent resulted in the isolation of compound **8** (87 mg). Fr.4 (32 g) was applied to silica gel CC (DCM: MeOH, 20:1–0:1) to yield 12 fractions (Fr.4.1–Fr.4.12). Semi-preparative HPLC was employed for the isolation of compound **9** (5 mg, t_R_ = 17 min) from Fr.4.7. **2** (11 mg, t_R_ = 12 min), **3** (9 mg, t_R_ = 13 min), and **4** (16 mg, t_R_ = 4.5 min) were obtained by isolating Fr.4.9 via semi-preparative HPLC. Fr.4.10 was purified by silica gel CC (PE: EA, 2.5:1), affording two compounds: **1** (5 mg) and **11** (6 mg). Compound **7** (4 mg) was obtained by separating Fr.4.11 using silica gel CC (PE: EA, 5:1).

### Spectroscopic data

*Sinomenoid A *(**1**). Colorless oil; $[\alpha ]_{D}^{20}$ − 21 (*c* 0.10, MeOH); UV (MeOH) *λ*_max_ (log *ε*) 280 (4.17) nm; IR (KBr) *ν*_max_ 3376, 2926, 1629, 1383, 1089, 981 cm^−1^; NMR data: Table 1; HRESIMS *m/z* 479.3488 [M + Na]^+^ (calcd for C_30_H_48_O_3_Na, 479.3501).

*Sinomenoid B *(**2**). Colorless oil; $[\alpha ]_{D}^{20}$ + 25 (*c* 0.10, MeOH); UV (MeOH) *λ*_max_ (log *ε*) 239 (4.07) nm; IR (KBr) *ν*_max_ 3373, 2946, 2866, 1450, 1382, 1034, 990 cm^−1^; NMR data: Table 1; HRESIMS *m/z* 443.3878 [M + H]^+^ (calcd for C_30_H_51_O_2_, 443.3889).

*Sinomenoid C *(**3**). Colorless oil; $[\alpha ]_{D}^{20}$ + 28 (*c* 0.10, MeOH); UV (MeOH) *λ*_max_ (log *ε*) 239 (4.37) nm; IR (KBr) *ν*_max_ 3475, 2941, 1447, 1382, 1067, 988 cm^−1^; NMR data: Table 1; HRESIMS *m/z* 443.3878 [M + H]^+^ (calcd for C_30_H_51_O_2_, 443.3889).

*(1R, 3S)−2, 3-Dihydro-1-hydroxy-3, 5, 6-trimethoxy-8-(2*′*-hydroxyethyl)−4(1H)-phenanthrenone *(**5**). Yellow bulk crystal; mp 154.8–156.5 °C; $[\alpha ]_{D}^{20}$ + 26 (*c* 0.10, MeOH); UV (MeOH) *λ*_max_ (log *ε*) 201 (4.38), 220 (4.56), 253 (4.40) nm; IR (KBr) *ν*_max_ 3397, 2946, 1602, 1424, 1343, 1261, 1108, 1037 cm^−1^; NMR data: Table 2; HRESIMS *m/z* 347.1485 [M + H]^+^ (calcd for C_19_H_23_O_6_, 347.1495).

*(1R, 3R)−2, 3-Dihydro-1-hydroxy-3, 5, 6-trimethoxy-8-(2*′*-hydroxyethyl)−4(1H)-phenanthrenone *(**6**). Yellow oil; $[\alpha ]_{D}^{20}$ + 31 (*c* 0.10, MeOH); UV (MeOH) *λ*_max_ (log *ε*) 202 (4.19), 220 (4.38), 254 (4.21) nm; IR (KBr) *ν*_max_ 3428, 2942, 1602, 1423, 1349, 1262, 1103, 1045 cm^−1^; NMR data: Table 2; HRESIMS *m/z* 369.1304 [M + Na]^+^ (calcd for C_19_H_22_O_6_Na, 369.1314).

11-Dehydroxy-dauricumidine (**13**). Colorless oil; $[\alpha ]_{D}^{20}$ + 73 (*c* 0.10, MeOH); UV (MeOH) λmax (log ε) 246 (4.42) nm; IR (KBr) *ν*_max_ 2948, 1690, 1455, 1429, 1362, 1324, 1270, 1244, 1192, 1141, 1062, 991 cm^−1^; NMR data: Table 3; HRESIMS *m/z* 368.1254 [M + H]^+^ (100) (calcd for C_18_H_23_NO_5_Cl, 368.1265).

### X-ray diffraction analysis

The structure of compound **5** (CCDC 2476411) was elucidated using SHELXT. The crystallographic data for this compound have been deposited at the CCDC and can be downloaded from https://www.ccdc.cam.ac.uk.

Crystallographic data for compound **5**: C_19_H_22_O_6_, *M* = 346.36, *a* = 10.6309(11) Å, *b* = 11.4283(9) Å, *c* = 14.1914(14) Å, *α* = 90°, *β* = 90°, *γ* = 90°, *V* = 1724.2(3)Å^3^, *T* = 301.48(10) K, space group P2_1_2_1_2_1_, *Z* = 4, *μ* (Cu K^α^) = 0.823 mm^−1^, 11,893 reflections collected, 3405 independent reflections (*R*_*int*_ = 0.0300, *R*_*sigma*_ = 0.0212). The final *R*_*1*_ values were 0.0500 (*I* > = *2σ* (*I*)) and *wR* (*F*^*2*^) values were 0.1394 (*I* > = *2σ* (*I*)). The final *R*_*1*_ values were 0.0591 (all data). Flack parameter = 0.11(11).

### NMR calculations

NMR calculations with DP4 + analysis were conducted for compounds **2** and **3** using published methods [30].

### ECD calculations

Preliminary geometries for six compounds (**1**–**3**, **6**, and **13**) were obtained using Chem3D software, and ECD calculations were carried out using established methods, as previously reported [31]. The calculated ECD spectra were obtained through SpecVis1.71 software by performing Boltzmann-weighting averaging of conformers with UV correction.

### Cell culture

RAW 264.7 cells were purchased from the China Center for Type Culture Collection. HeLa/PTX cells were established in our laboratory as reported previously [32]. All cells were maintained in DMEM containing 10% FBS and 1% P/S at 37 °C with 5% CO_2_.

### Anti-inflammatory assay

RAW 264.7 macrophages were stimulated with LPS (1 μg/mL) in combination with different isolates for 24 h. NO production was subsequently assessed using Griess reagents as previously described [14].

### Molecular docking and dynamics simulations analysis

The X-ray crystal structure of iNOS (PDB ID: 3E6T) was obtained from the Protein Data Bank (https://www.rcsb.org/). AutoDock Vina 1.5.6 was employed to perform docking calculations [33]. The docked complexes were then examined and rendered using Discovery Studio 4.5 [34] and PyMOL 3.0 software.

GROMACS 2020.6 was employed to conduct molecular dynamics (MD) simulations. In the system, TIP3P was chosen as the water model, and charge neutralization was achieved by adding Na^+^ and Cl^−^. Prior to the simulations, energy minimization was performed first, followed by pre-equilibration steps under NVT and NPT conditions. Finally, MD simulations were performed with a total simulation time of 50 ns.

### Cell viability assay

HeLa/PTX cells (5 × 10^3^ cells/well) were treated with drugs for 72 h. Following treatment, cell viability was measured using the MTT assay.

### Colony formation assay

HeLa/PTX cells were treated with drugs for 12 h, then cultured without drug for 14 days as previously described [35]. Viable colonies were stained and quantified.

### Nuclear morphology assay

After drug treatment, cell staining was performed using Hoechst 33,258 as previously described [32].

### Western blot assay

Samples were processed using 10% SDS-PAGE and then transferred to PVDF. After blocking, the membrane was incubated sequentially with primary antibodies overnight and with secondary antibodies for 2 h. A chemiluminescence system was used to visualize the image.

### Statistical analysis

Data are shown as the mean ± SD (n = 3). Statistical analyses were performed using GraphPad Prism 8.3.0 and Origin 2024. One-way ANOVA followed by Tukey’s multiple comparisons test was employed to evaluate statistical significance (*P* < 0.05).

## Supplementary Information

Supplementary Material 1

## Data Availability

The data generated in this study were included in the article and its supplementary materials.
