# Supplementary material for: Bioactive components from Sinomenium acutum exhibit anti-inflammatory activity and sensitize cervical cancer cells to paclitaxel
Source: Nat Prod Bioprospect. 2026 Oct 19;16(1):94. doi: 10.1007/s13659-026-00652-w (PMC13680603; doi:10.1007/s13659-026-00652-w)
Supplement: Supplementary file 1 [file 13659_2026_652_MOESM1_ESM.docx]

**Supporting Information**

**Bioactive components from *Sinomenium acutum* exhibit anti-inflammatory activity and sensitize cervical cancer cells to paclitaxel**

Xilei Wang ^a,b^, Chengjing Zhu ^a,b^, Xinyu Du ^a,b^, Qiang Wang ^c^, Jianqing Yu ^a,b,^*

^a^ Department of Pharmacy, Zhongnan Hospital of Wuhan University, School of Pharmaceutical Sciences, Wuhan University, Wuhan 430071, China

^b^ Key Laboratory of Combinatorial Biosynthesis and Drug Discovery (Wuhan University), Ministry of Education, Wuhan University School of Pharmaceutical Sciences, Wuhan 430071, China.

^c^ College of Pharmacy, South-Central Minzu University, 182 Minzu Road, Wuhan, 430074, China.

***Correspondence:**

Jianqing Yu

*E-mail address:* [jqyu@](mailto:jqyu@)whu.edu.cn.

**Contents**

**Fig. S1** ^1^H NMR spectrum of compound **1** in CDCl_3_ (400 MHz)

**Fig. S2** ^13^C NMR spectrum of compound **1** in CDCl_3_ (100 MHz)

**Fig. S3** HSQC spectrum of compound **1** in CDCl_3_

**Fig. S4** HMBC spectrum of compound **1** in CDCl_3_

**Fig. S5** ^1^H–^1^H COSY spectrum of compound **1** in CDCl_3_

**Fig. S6** NOESY spectrum of compound **1** in CDCl_3_

**Fig. S7** HRESIMS spectrum of compound **1**

**Fig. S8** UV spectrum of compound **1**

**Fig. S9** IR spectrum of compound **1**

**Fig. S10** ^1^H NMR spectrum of compound **2** in CDCl_3_ (400 MHz)

**Fig. S11** ^13^C NMR spectrum of compound **2** in CDCl_3_ (100 MHz)

**Fig. S12** HSQC spectrum of compound **2** in CDCl_3_

**Fig. S13** HMBC spectrum of compound **2** in CDCl_3_

**Fig. S14** ^1^H–^1^H COSY spectrum of compound **2** in CDCl_3_

**Fig. S15** NOESY spectrum of compound **2** in CDCl_3_

**Fig. S16** HRESIMS spectrum of compound **2**

**Fig. S17** UV spectrum of compound **2**

**Fig. S18** IR spectrum of compound **2**

**Fig. S19** 1H NMR spectrum of compound **3** in CDCl_3_ (400 MHz)

**Fig. S20** ^13^C NMR spectrum of compound **3** in CDCl_3_ (100 MHz)

**Fig. S21** HSQC spectrum of compound **3** in CDCl_3_

**Fig. S22** HMBC spectrum of compound **3** in CDCl_3_

**Fig. S23** ^1^H–^1^H COSY spectrum of compound **3** in CDCl_3_

**Fig. S24** NOESY spectrum of compound **3** in CDCl_3_

**Fig. S25** HRESIMS spectrum of compound **3**

**Fig. S26** UV spectrum of compound **3**

**Fig. S27** IR spectrum of compound **3**

**Fig. S28** ^1^H NMR spectrum of compound **5** in CDCl_3_ (400 MHz)

**Fig. S29** ^13^C NMR spectrum of compound **5** in CDCl_3_ (100 MHz)

**Fig. S30** HSQC spectrum of compound **5** in CDCl_3_

**Fig. S31** HMBC spectrum of compound **5** in CDCl_3_

**Fig. S32** ^1^H–^1^H COSY spectrum of compound **5** in CDCl_3_

**Fig. S33** NOESY spectrum of compound **5** in CDCl_3_

**Fig. S34** HRESIMS spectrum of compound **5**

**Fig. S35** UV spectrum of compound **5**

**Fig. S36** IR spectrum of compound **5**

**Fig. S37** ^1^H NMR spectrum of compound **6** in CDCl_3_ (400 MHz)

**Fig. S38** ^13^C NMR spectrum of compound **6** in CDCl_3_ (100 MHz)

**Fig. S39** HSQC spectrum of compound **6** in CDCl_3_

**Fig. S40** HMBC spectrum of compound **6** in CDCl_3_

**Fig. S41** ^1^H–^1^H COSY spectrum of compound **6** in CDCl_3_

**Fig. S42** NOESY spectrum of compound **6** in CDCl_3_

**Fig. S43** HRESIMS spectrum of compound **6**

**Fig. S44** UV spectrum of compound **6**

**Fig. S45** IR spectrum of compound **6**

**Fig. S46** ^1^H NMR spectrum of compound **13** in CD_3_OD (400 MHz)

**Fig. S47** ^13^C NMR spectrum of compound **13** in CD_3_OD (100 MHz)

**Fig. S48** HSQC spectrum of compound **13** in CD_3_OD

**Fig. S49** HMBC spectrum of compound **13** in CD_3_OD

**Fig. S50** ^1^H–^1^H COSY spectrum of compound **13** in CD_3_OD

**Fig. S51** NOESY spectrum of compound **13** in CD_3_OD

**Fig. S52** HRESIMS spectrum of compound **13**

**Fig. S53** UV spectrum of compound **13**

**Fig. S54** IR spectrum of compound **13**

**Table S1.** NMR computation of **2** with DP4+ analysis for 20R*, 20S*

**Table S2.** NMR computation of **3** with DP4+ analysis for 20R*, 20S*

**Table S3.** NMR computation of **5** with DP4+ analysis for 1R*,3S* and 1R*,3R*

**Table S4.** NMR computation of **6** with DP4+ analysis for 1R*,3S* and 1R*,3R*

**Table S5.** Crystal data and structure refinement for **5**

**Table S6.** Fractional Atomic Coordinates (×10^4^) and Equivalent Isotropic Displacement Parameters (Å^2^×10^3^) for **5**. Ueq is defined as 1/3 of the trace of the orthogonalised UIJ tensor

**Table S7.** Bond Lengths for **5**

**Table S8.** Bond Angles for **5**

**Table S9.** Optical rotation data and specific rotation calculation of diastereomers **5** and **6**

**Fig. S1** ^1^H NMR spectrum of compound **1** in CDCl_3_ (400 MHz)

**Fig. S2** ^13^C NMR spectrum of compound **1** in CDCl_3_ (100 MHz)

**Fig. S3** HSQC spectrum of compound **1** in CDCl_3_

**Fig. S4** HMBC spectrum of compound **1** in CDCl_3_

Magnified partial views of **Fig. S4**

**Fig. S5** ^1^H–^1^H COSY spectrum of compound **1** in CDCl_3_

**Fig. S6** NOESY spectrum of compound **1** in CDCl_3_

**Fig. S7** HRESIMS spectrum of compound **1**

**Fig. S8** UV spectrum of compound **1**

**Fig. S9** IR spectrum of compound **1**

**Fig. S10** ^1^H NMR spectrum of compound **2** in CDCl_3_ (400 MHz)

**Fig. S11** ^13^C NMR spectrum of compound **2** in CDCl_3_ (100 MHz)

**Fig. S12** HSQC spectrum of compound **2** in CDCl_3_

**Fig. S13** HMBC spectrum of compound **2** in CDCl_3_

**Fig. S14** ^1^H–^1^H COSY spectrum of compound **2** in CDCl_3_

**Fig. S15** NOESY spectrum of compound **2** in CDCl_3_

**Fig. S16** HRESIMS spectrum of compound **2**

**Fig. S17** UV spectrum of compound **2**

**Fig. S18** IR spectrum of compound **2**

**Fig. S19** ^1^H NMR spectrum of compound **3** in CDCl_3_ (400 MHz)

**Fig. S20** ^13^C NMR spectrum of compound **3** in CDCl_3_ (100 MHz)

**Fig. S21** HSQC spectrum of compound **3** in CDCl_3_

**Fig. S22** HMBC spectrum of compound **3** in CDCl_3_

**Fig. S23** ^1^H–^1^H COSY spectrum of compound **3** in CDCl_3_

**Fig. S24** NOESY spectrum of compound **3** in CDCl_3_

**Fig. S25** HRESIMS spectrum of compound **3**

**Fig. S26** UV spectrum of compound **3**

**Fig. S27** IR spectrum of compound **3**

**Fig. S28** ^1^H NMR spectrum of compound **5** in CDCl_3_ (400 MHz)

**Fig. S29** ^13^C NMR spectrum of compound **5** in CDCl_3_ (100 MHz)

**Fig. S30** HSQC spectrum of compound **5** in CDCl_3_

**Fig. S31** HMBC spectrum of compound **5** in CDCl_3_

**Fig. S32** ^1^H–^1^H COSY spectrum of compound **5** in CDCl_3_

**Fig. S33** NOESY spectrum of compound **5** in CDCl_3_

**Fig. S34** HRESIMS spectrum of compound **5**

**Fig. S35** UV spectrum of compound **5**

**Fig. S36** IR spectrum of compound **5**

**Fig. S37** ^1^H NMR spectrum of compound **6** in CDCl_3_ (400 MHz)

**Fig. S38** ^13^C NMR spectrum of compound **6** in CDCl_3_ (100 MHz)

**Fig. S39** HSQC spectrum of compound **6** in CDCl_3_

**Fig. S40** HMBC spectrum of compound **6** in CDCl_3_

**Fig. S41** ^1^H–^1^H COSY spectrum of compound **6** in CDCl_3_

**Fig. S42** NOESY spectrum of compound **6** in CDCl_3_

**Fig. S43** HRESIMS spectrum of compound **6**

**Fig. S44** UV spectrum of compound **6**

**Fig. S45** IR spectrum of compound **6**

**Fig. S46** ^1^H NMR spectrum of compound **13** in CD_3_OD (400 MHz)

**Fig. S47** ^13^C NMR spectrum of compound **13** in CD_3_OD (100 MHz)

**Fig. S48** HSQC spectrum of compound **13** in CD_3_OD

**Fig. S49** HMBC spectrum of compound **13** in CD_3_OD

**Fig. S50** ^1^H–^1^H COSY spectrum of compound **13** in CD_3_OD

**Fig. S51** NOESY spectrum of compound **13** in CD_3_OD

**Fig. S52** HRESIMS spectrum of compound **13**

**Fig. S53** UV spectrum of compound **13**

**Fig. S54** IR spectrum of compound **13**

**Table S1** NMR computation of **2** with DP4+ analysis for 20R*, 20S*.

**Table S2** NMR computation of **3** with DP4+ analysis for 20R*, 20S*.

**Table S3** NMR computation of **5** with DP4+ analysis for 1R*,3S* and 1R*,3R*.

**Table S4** NMR computation of **6** with DP4+ analysis for 1R*,3S* and 1R*,3R*.

| Table **S5** Crystal data and structure refinement for **5**. | |
| --- | --- |
| Empirical formula | C_19_H_22_O_6_ |
| Formula weight | 346.36 |
| Temperature/K | 301.48 |
| Crystal system | orthorhombic |
| Space group | P2_1_2_1_2_1_ |
| a/Å | 10.6309(11) |
| b/Å | 11.4283(9) |
| c/Å | 14.1914(14) |
| α/° | 90 |
| β/° | 90 |
| γ/° | 90 |
| Volume/Å^3^ | 1724.2(3) |
| Z | 4 |
| ρ_calc_g/cm^3^ | 1.334 |
| μ/mm^‑1^ | 0.823 |
| Shape | block |
| Crystal size/mm^3^ | 0.26 × 0.22 × 0.12 |
| Radiation type | CuKα |
| Independent reflections | 3405 |
| GooF | 1.149 |
| Parameters | 232 |
| Restraints | 0 |
| Largest peak | 0.245 |
| Deepest hole | -0.209 |
| Hooft parameter | 0.22(7) |
| Flack parameter | 0.11(11) |

| **Table S6** Fractional Atomic Coordinates (×10^4^) and Equivalent Isotropic Displacement Parameters (Å2×10^3^) for **5**. Ueq is defined as 1/3 of the trace of the orthogonalised UIJ tensor. | | | | |
| --- | --- | --- | --- | --- |
| **Atom** | ***x*** | ***y*** | ***z*** | **U(eq)** |
| O1 | 2757(3) | -1496(2) | 7228(2) | 71.6(8) |
| O2 | 2010(3) | 572(2) | 6548.6(19) | 62.4(7) |
| O3 | 5532(4) | -782(3) | 4661(2) | 90.4(11) |
| O4 | 2778(3) | 2343(2) | 7741.9(18) | 63.1(7) |
| O5 | 2249(3) | 4682.5(19) | 7750(2) | 72.2(8) |
| O6 | 6265(3) | 6849(2) | 4532(2) | 77.4(9) |
| C1 | 2221(6) | -1207(4) | 8118(4) | 86.7(14) |
| C2 | 3701(4) | -708(3) | 6927(3) | 60.5(9) |
| C3 | 4362(5) | -1237(3) | 6077(3) | 71.5(11) |
| C4 | 4984(4) | 851(3) | 5616(3) | 60.2(9) |
| C5 | 4062(3) | 1293(3) | 6198(3) | 53.3(8) |
| C6 | 3159(3) | 434(3) | 6601(2) | 53.0(8) |
| C7 | 5642(4) | 1613(3) | 5005(3) | 70.3(11) |
| C8 | 5352(4) | 2778(3) | 4972(3) | 67.9(10) |
| C9 | 4455(4) | 3287(3) | 5599(3) | 56.4(8) |
| C10 | 3855(3) | 2543(3) | 6272(2) | 51.3(8) |
| C11 | 3125(3) | 3045(3) | 6997(2) | 52.6(8) |
| C12 | 2915(4) | 4246(3) | 7011(3) | 55.0(8) |
| C13 | 3407(4) | 4948(3) | 6293(3) | 60.3(9) |
| C14 | 4181(4) | 4508(3) | 5597(3) | 57.9(8) |
| C15 | 4707(4) | 5359(3) | 4872(3) | 64.9(10) |
| C16 | 5932(4) | 5926(3) | 5167(3) | 64.7(10) |
| C17 | 2035(6) | 5909(4) | 7780(4) | 107(2) |
| C18 | 1477(5) | 2318(4) | 8000(4) | 85.8(15) |
| C19 | 5328(4) | -446(3) | 5607(3) | 65.3(9) |

**Table S7** Bond Lengths for **5**.

| **Atom** | **Atom** | **Length/Å** |
| --- | --- | --- |
| O1 | C1 | 1.424(5) |
| O1 | C2 | 1.415(5) |
| O2 | C6 | 1.234(4) |
| O3 | C19 | 1.413(5) |
| O4 | C11 | 1.378(4) |
| O4 | C18 | 1.431(5) |
| O5 | C12 | 1.360(4) |
| O5 | C17 | 1.420(5) |
| O6 | C16 | 1.432(5) |
| C2 | C3 | 1.520(6) |
| C2 | C6 | 1.499(4) |
| C3 | C19 | 1.523(6) |
| C4 | C5 | 1.378(5) |
| C4 | C7 | 1.414(6) |
| C4 | C19 | 1.527(5) |
| C5 | C6 | 1.487(5) |
| C5 | C10 | 1.449(4) |
| C7 | C8 | 1.367(5) |
| C8 | C9 | 1.428(6) |
| C9 | C10 | 1.429(5) |
| C9 | C14 | 1.425(5) |
| C10 | C11 | 1.411(5) |
| C11 | C12 | 1.391(4) |
| C12 | C13 | 1.398(5) |
| C13 | C14 | 1.381(5) |
| C14 | C15 | 1.522(5) |
| C15 | C16 | 1.513(6) |

**Table S8** Bond Angles for **5**

| **Atom** | **Atom** | **Atom** | **Angle/˚** |
| --- | --- | --- | --- |
| C2 | O1 | C1 | 113.9(3) |
| C11 | O4 | C18 | 117.8(3) |
| C12 | O5 | C17 | 117.9(3) |
| O1 | C2 | C3 | 108.3(3) |
| O1 | C2 | C6 | 112.0(3) |
| C6 | C2 | C3 | 106.2(3) |
| C2 | C3 | C19 | 115.0(3) |
| C5 | C4 | C7 | 119.6(3) |
| C5 | C4 | C19 | 122.0(3) |
| C7 | C4 | C19 | 118.3(4) |
| C4 | C5 | C6 | 116.6(3) |
| C4 | C5 | C10 | 120.8(3) |
| C10 | C5 | C6 | 121.7(3) |
| O2 | C6 | C2 | 120.7(3) |
| O2 | C6 | C5 | 122.1(3) |
| C5 | C6 | C2 | 116.4(3) |
| C8 | C7 | C4 | 120.5(4) |
| C7 | C8 | C9 | 121.8(3) |
| C8 | C9 | C10 | 118.1(3) |
| C14 | C9 | C8 | 122.3(3) |
| C14 | C9 | C10 | 119.5(3) |
| C9 | C10 | C5 | 118.1(3) |
| C11 | C10 | C5 | 122.5(3) |
| C11 | C10 | C9 | 119.4(3) |
| O4 | C11 | C10 | 118.0(3) |
| O4 | C11 | C12 | 121.4(3) |
| C12 | C11 | C10 | 120.0(3) |
| O5 | C12 | C11 | 117.2(3) |
| O5 | C12 | C13 | 123.2(3) |
| C11 | C12 | C13 | 119.7(3) |
| C14 | C13 | C12 | 122.4(3) |
| C9 | C14 | C15 | 123.5(4) |
| C13 | C14 | C9 | 118.4(3) |
| C13 | C14 | C15 | 118.1(3) |
| C16 | C15 | C14 | 113.8(3) |
| O6 | C16 | C15 | 110.7(4) |
| O3 | C19 | C3 | 111.1(4) |
| O3 | C19 | C4 | 108.0(3) |
| C3 | C19 | C4 | 114.3(3) |

**Table S9.** Optical rotation data and specific rotation calculation of diastereomers **5** and **6**

| Compound | *α* (°) | *c* (g/mL) | *l* (dm) | Raw calculated ${\text{[}\text{α}\text{]}}_{\text{D}}^{\text{t}}$ | Rounded values |
| --- | --- | --- | --- | --- | --- |
| **5** | +0.255 | 0.001 | 10 | +25.5 | +26 |
| **6** | +0.309 | 0.001 | 10 | +30.9 | +31 |

Note: All specific rotation values in the main text are rounded to integers.

Optical rotations were determined on a PerkinElmer Model 341 polarimeter.

Calculation formula: ${\text{[}\text{α}\text{]}}_{\text{D}}^{\text{t}}$= $\frac{\text{α}}{\text{cl}}$

where *α* = observed rotation angle (°); *c* = sample concentration (g/mL); *l* = length of polarimeter tube (dm); *D* = sodium D-line (589 nm); *t* = measurement temperature (°C).

Measurement parameters: *c* = 0.001 g/mL, *l* =10 dm, *t* = 20 °C
